# Contribution of ionic interactions to stationary phase selectivity in hydrophilic interaction chromatography

**DOI:** 10.1002/jssc.202200165

**Published:** 2022-04-07

**Authors:** Martin Gilar, Kenneth D. Berthelette, Thomas H. Walter

**Affiliations:** ^1^ Separations R&D Waters Corporation Milford Massachusetts USA

**Keywords:** column selectivity, hydrophilic interaction chromatography, ionic interactions, mixed‐mode stationary phases, similarity factors

## Abstract

We compared the separation selectivities of 19 different hydrophilic interaction chromatography columns. The stationary phases included underivatized silica and hybrid particles, cyano‐bonded silica, materials with neutral ligands such as amide, diol, pentahydroxy, and urea, zwitterionic sorbents, and mixed‐mode materials with amine functionalities. A set of 77 small molecules was used to evaluate the columns. We visualized the retention behavior of the different columns using retention time correlation plots. The analytes were classified as cations, anions, or neutral based on their estimated charge under the separation conditions. This involved adjusting the dissociation constants of the analytes for the acetonitrile content of the mobile phase and experimentally determining the pH of the mobile phase containing 70% acetonitrile. The retention correlation plots show that the selectivity differences strongly depended on ionic interactions. Comparisons of the neutral stationary phases (e.g., diol vs. amide) showed more similar selectivity than did comparisons of neutral columns versus columns with cation or anion exchange activity (bare silica or amine columns, respectively). The zwitterionic columns did not behave as perfectly neutral. The correlation plots indicated that they exhibited either cation or anion exchange activity, although to a lesser degree than the silica and amine‐containing stationary phases.

**Abbreviations**
Amio: amiodarone
BEH: ethylene‐bridged hybrid
Bupro: bupropion
Ceti: cetirizine
Cyto: cytosine
Epica: epicatechin
ESI: electrospray ionization
Furo: furosemide
Lido: lidocaine
LSER: linear solvation energy relationships
Prilo: prilocaine
QSM: quaternary solvent manager
r2: correlation coefficient
s2: selectivity parameter
s: selectivity factor
z: charge
5F‐Cyto: 5‐fluorocytosine

## 1 INTRODUCTION

Hydrophilic interaction chromatography (HILIC) is a separation method that uses a polar stationary phase and a less polar mobile phase typically containing a mixture of ACN and an aqueous buffer [1, 2]. It provides greater retention of polar analytes than RP chromatography, as well as higher sensitivity when using electrospray MS detection [3, 4, 5]. These attributes have proven to be beneficial for the separations of a wide range of analyte classes, including oligonucleotides, amino acids, peptides, glycans, pharmaceuticals, and cellular metabolites [6, 7, 8, 9, 10, 11, 12]. Over the last two decades, a number of HILIC stationary phases have been introduced [2, 13–18]. Among these, the most cited chemistries are unbonded silica, zwitterionic, amide, diol, and amino materials [18].

The primary retention mechanism in HILIC is believed to be the partitioning of analytes into the water‐rich layer that forms at the stationary phase surface [1, 2, 19–23]. However, other mechanisms also participate, most importantly electrostatic and hydrogen bonding interactions [24, 25, 26, 27]. While the principles of how to control retention and selectivity in HILIC have been well documented [28, 29, 30], the selection of the most suitable column for a specific application remains largely a trial‐and‐error task.

Several studies have compared the selectivity of different HILIC stationary phases. McCalley reported a comparison of the selectivity of five HILIC stationary phases for a mixture of eight compounds including acids, bases, and neutrals [31]. The mobile phases contained a ${}_{w}^{w}\mathrm{pH}$ 3.0 ammonium formate buffer and different buffer concentrations were used to assess the contribution of ionic interactions. The results demonstrated that the protonated bases studied showed considerable ionic interactions, not only for an unbonded silica material but also for three of the bonded silica stationary phases.

Dinh et al. investigated the selectivities of 21 commercially available HILIC columns using a set of 21 test solutes that were chosen to probe specific interactions, including anion and cation exchange, hydrogen bonding, dipole‐dipole, quadrupolar electrostatic, π‐ π, and shape selectivity [24]. Isocratic separations were carried out using an 80/20 v/v ACN/25 mM aqueous ammonium acetate (${}_{w}^{w}\mathrm{pH}$ 6.8) mobile phase. Principal component analysis was used to compare the columns based on the separation factors for 17 pairs of analytes. The results showed that the columns fell into four groups: (i) cation exchange (unbonded silicas), (ii) anion exchange (amino materials), (iii) dipole‐dipole and multipoint hydrogen bonding (polymeric sulfobetaine and poly(2‐sulfoethylaspartamide) stationary phases and (iv) low specific interaction (hydroxyl, diol, amide, and monomeric zwitterionic materials). This study confirmed that partitioning is the dominant mechanism, with ion‐exchange interactions providing “a powerful and orthogonal selectivity factor” for charged solutes.

Ikegami and coworkers characterized 45 different HILIC columns using a mobile phase containing a 90/10 v/v ACN/20 mM aqueous ammonium acetate (${}_{w}^{w}\mathrm{pH}$ 4.7) mobile phase and thirteen analytes were chosen to interrogate different types of selectivity [32, 33]. This included selectivity for hydroxy groups, methyl groups, regio, and configurational isomers, molecular shape, and for an anion and a cation. Principal component analysis was used to classify the stationary phases based on the similarity of their relative retentions [33]. The results suggested that ion‐exchange interactions were the predominant components. Removing the ion‐exchange terms from the data set revealed more subtle effects, such as the differences in hydroxy group selectivity associated with polymer‐functionalized versus silane‐bonded silicas.

Fountain and coworkers compared the retention factors of 28 acidic, basic and neutral analytes on five different columns (three commercially available and two experimental prototypes) using mobile phases containing either a ${}_{w}^{w}\mathrm{pH}$ 3 (ammonium formate) or a ${}_{w}^{w}\mathrm{pH}$ 9 (ammonium hydroxide) buffer [34]. Following an approach described for RP separations by Neue et al. [35], selectivity differences were calculated by plotting the retention factors for pairs of columns, then calculating selectivity parameters (s^2^) from the correlation coefficients (r^2^):

(1) $$s^{2}=1-r^{2}$$

The s^2^ values calculated for the different stationary phases were larger than those reported for RP stationary phases, with values as large as 0.948.

Vlčková *et al* reported a comparison of the retention behavior for eight different commercially available HILIC columns and 35 analytes [36]. Isocratic separations were carried out using ${}_{w}^{w}\mathrm{pH}$ 3.8 and 6.8 ammonium acetate buffers. The selectivity differences for certain pairs of columns were calculated using Neue's method, except that the s^2^ values were determined separately for neutrals, nucleotide bases, other bases, and acids. The largest values (0.8671–0.9993) were found when comparing an aminopropyl column to unbonded silica, unbonded ethylene‐bridged hybrid (BEH), and cyanopropyl columns for the acidic compounds at pH 3.8. Large values (0.9472–0.9928) were also obtained for the nucleotide bases comparing the aminopropyl column to diol, amide, and cyanopropyl columns at pH 3.8. These results further demonstrate the importance of ionic interactions when comparing the selectivities of different HILIC stationary phases.

Schuster and Lindner investigated retention mechanisms in HILIC using linear solvation energy relationships (LSER) [37, 38]. They tested 22 different stationary phases, half being commercially available and half being research materials. Using isocratic separations with mobile phases containing either a ${}_{w}^{w}\mathrm{pH}$ 3 ammonium formate buffer or a ${}_{w}^{w}\mathrm{pH}$ 5 ammonium acetate buffer, retention factors were measured for 68 analytes. The LSER equation used to analyze the results contained terms for the ionic contributions to retention. Hierarchical Cluster Analysis was used to group the stationary phases based on the similarity of the retention factors. The ionization behavior of the stationary phases (acidic, basic, or neutral) was a major determinant of the grouping of the data. It was noted that a major limitation of using LSER in HILIC is that the solute descriptors are only available for solutes in their neutral form, while many solutes of interest are ionized under typical HILIC separation conditions.

In an alternative approach, Wang and coworkers developed a hydrophilic‐subtraction model to characterize and compare HILIC columns [27]. The model included terms for hydrophilic partitioning, hydrogen‐bond donor, hydrogen‐bond acceptor, anion exchange, and cation exchange contributions to the log of the retention factors. Using a set of 41 analytes comprised of acidic, basic, and neutral molecules and isocratic separations employing a mobile phase containing a ${}_{w}^{w}\mathrm{pH}$ 3.3 ammonium formate buffer, retention factors were measured for eight different columns. These results were used to calculate solute and column descriptors. The solute descriptors were then used to obtain column parameters for 15 additional columns. The hydrophilic‐subtraction model gave better correlation coefficients than the LSER approach. To compare the selectivities of the complete set of 23 stationary phases, the column parameters were used to calculate the angles between the solvation energy vectors for pairs of columns. The largest angles, indicating the greatest differences, were found between cation‐exchange and anion‐exchange stationary phases.

To expand on these prior studies, we evaluated 19 commercially available HILIC columns, including unbonded silica, cyano, diol, pentahydroxy, urea, amide, zwitterionic, and mixed‐mode chemistries on either silica or BEH organic/inorganic particles. A number of these columns were not included in previous comparisons of column selectivity. Seventy‐seven analytes were used, including acidic, basic, zwitterionic, and neutral species. The goal of this study was to investigate the contribution of electrostatic interactions to HILIC separation selectivity. This required consideration of the effect of the ACN content on the mobile phase pH and the dissociation constants of the analytes.

## 2 MATERIALS AND METHODS

### 2.1 Chemicals

LC‐MS grade ACN, and MS grade acetic acid were purchased from Fisher Scientific (Hampton, NH, USA). Ammonium acetate (> 99.995%), and all test compounds were purchased from Millipore‐Sigma (Burlington, MA, USA). Deionized water was produced using a Millipore Milli‐Q™ system (Burlington, MA, USA).

### 2.2 Instrumentation and columns

Tests were performed on an ACQUITY UPLC H‐Class Instrument from Waters Corporation (Milford, MA, USA) consisting of a quaternary solvent manager, a sample manager with a flow‐through needle, a column manager with column auxiliary utilizing active pre‐heaters, a photodiode array detector, and either a single quadrupole mass detector (ACQUITY QDa Detector) or a tandem quadrupole mass spectrometer (XEVO TQD). The mass spectrometers were set to full scan in both ESI+ and ESI‐ modes with a mass range of 50–1000 Da. Data processing and peak identification were performed using extracted ion chromatograms of the target analytes.

**FIGURE 1:**
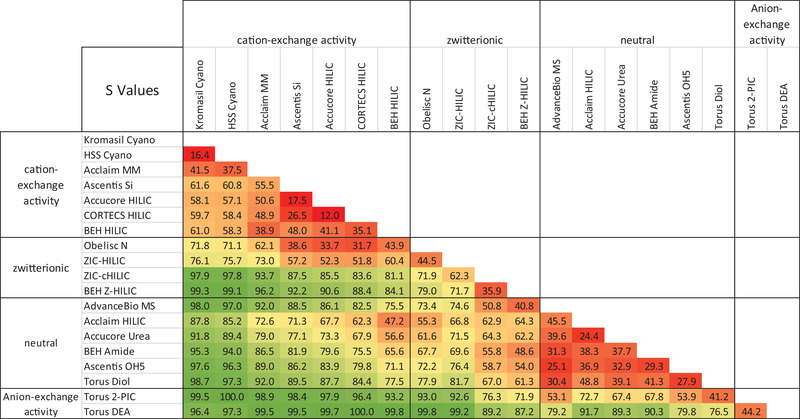
Selectivity values (s) for pairwise comparisons of the 19 columns evaluated. The cells are shaded to indicate the magnitude of the s values, ranging from red for the smallest values to green for the largest

All columns tested were 2.1 × 150 mm and contained particles with sizes ranging from 2.5 to 5 μm. XBridge BEH HILIC, XBridge BEH Amide, CORTECS HILIC, Torus DEA, Torus Diol, XSelect HSS Cyano, and Atlantis Premier BEH Z‐HILIC columns were from Waters (Milford, MA, USA). Acclaim Mixed Mode HILIC‐1, Acclaim HILIC‐10, Accucore HILIC, and Accucore Urea columns were purchased from Thermo Fisher (Hampton, NH, USA). SeQuant ZIC‐HILIC, SeQuant ZIC‐cHILIC, Kromasil Cyano, Ascentis Si, and Ascentis Express OH5 columns were purchased from Millipore‐Sigma (Burlington, MA, USA). An AdvanceBio MS Spent Media column was purchased from Agilent Technologies (Santa Clara, CA, USA) and an Obelisc N column was obtained from SiELC Technologies (Wheeling, IL, USA). Out of 19 columns used in this study, seven have cation‐exchange activity, four are zwitterionic, six can be classified as neutral and two have anion‐exchange activity (for details see Figure 1). Column description is given in Table 1; the manufacturer suggested ${}_{w}^{w}\mathrm{pH}$ stability ranges are not shown. More relevant ${}_{w}^{s}\mathrm{pH}$ stability guidelines should be used in HILIC as discussed in recent publication [39].

**TABLE 1:** Description of the 19 columns used in this study. The columns are divided into the following groups: Columns with cation‐exchange activity, zwitterionic columns, neutral columns, and columns with anion exchange activity

**Column name**	**Column description**	**Manufacturer**	**Separation type**	**Functional ligand**	**Particle type**	**Particle size (μm)**
Kromasil Cyano	Kromasil Cyano	Kromasil	HILIC‐CX	cyano	fully porous silica	5.0
HSS Cyano	HSS Cyano	Waters Corp	HILIC‐CX	cyano	fully porous silica	2.5
Acclaim Mixed Mode HILIC‐1	Acclaim MM	ThermoFisher Scientific	HILIC‐CX‐RP	alkyl chain with diol terminus	fully porous silica	3.0
Ascentis Si	Ascentis Si	Ascentis	HILIC‐CX	unbonded silica	fully porous silica	3.0
Accucore HILIC	Accucore HILIC	ThermoFisher Scientific	HILIC‐CX	unbonded silica	solid‐sore silica	2.6
CORTECS HILIC	CORTECS HILIC	Waters	HILIC‐CX	unbonded silica	solid‐sore silica	2.7
XBridge BEH HILIC	BEH HILIC	Waters	HILIC‐CX	unbonded hybrid silica	fully porous hybrid	2.5
Sielc Obelisc N	Obelisc N	Sielc	HILIC‐Zwitterion	proprietary hydrophilic CX AX	fully porous silica	5.0
SeQuant ZIC‐HILIC	ZIC‐HILIC	Sequant	HILIC‐Zwitterion	sulfobetaine	fully porous silica	3.5
SeQuant ZIC‐cHILIC	ZIC‐cHILIC	Sequant	HILIC‐Zwitterion	phosphorylcholine	fully porous silica	3.0
Atlantis Premier BEH Z‐HILIC	BEH Z‐HILIC	Waters	HILIC‐Zwitterion	sulfobetaine	fully porous hybrid	2.5
AdvanceBio MS Spent Media	AdvanceBio MS	Agilent	HILIC‐Zwitterion	proprietary zwitterion	solid‐sore hybrid silica	2.7
Acclaim HILIC‐10	Acclaim HILIC	ThermoFisher Scientific	HILIC‐Neutral	proprietary hydrophilic	fully porous silica	3.0
Accucore Urea	Accucore Urea	ThermoFisher Scientific	HILIC‐Neutral	urea	solid‐sore silica	3.0
XBridge BEH Amide	BEH Amide	Waters	HILIC‐Neutral	amide	fully porous hybrid	2.5
Ascentis Express OH5	Ascentis OH5	Ascentis	HILIC‐Neutral	pentahydroxy	solid‐sore silica	2.7
Torus Diol	Torus Diol	Waters	HILIC‐Neutral	diol	fully porous hybrid	5.0
Torus 2‐PIC	Torus 2‐PIC	Waters	HILIC‐AX	2‐picolylamine	fully porous hybrid	5.0
Torus DEA	Torus DEA	Waters	HILIC‐AX	diethylamine	fully porous hybrid	5.0

Abbreviation: HILIC, hydrophilic interaction chromatography.

### 2.3 Sample and mobile phase preparation

The structures of the 77 analytes, the mass to charge ratios used for MS detection, pK_a_ values (ChemAxon values from the DrugBank database), and calculated charges are provided in Table S1. ChemAxon software online subscription was purchased from chemicalize.com, 2022. Stock solutions of each analyte were prepared at a concentration of 1.0 mg/mL in ACN:water (95:5 v/v). Ten sample mixtures were created by combining up to 10 test probes. For sample mixtures with less than 10 probes appropriate volumes of 95:5 ACN:water v/v were added so that the final sample concentration for all test probes was 0.1 mg/mL. Test mixtures were created with care to avoid the presence of isobaric compounds in the same mixture. This allowed for peak identification by mass to charge ratio and reduced the potential for misidentification. The aqueous buffer used in the mobile phase was prepared by dissolving ammonium acetate in Milli‐Q water, then adjusting the ${}_{w}^{w}\mathrm{pH}$ to 5.0 ± 0.1 with acetic acid. The mobile phases were mixed using the quaternary solvent manager, by blending ACN, water, and 200 mM aqueous buffer.

The ${}_{w}^{s}\mathrm{pH}$ value of the composition corresponding approximately to the midpoint of the mobile phase gradient was measured as follows. The pH meter was calibrated using aqueous buffers. Then 200 mM aqueous ammonium acetate buffer, ${}_{w}^{w}\mathrm{pH}$ 5, was mixed with water and ACN in a 5:25:70 v:v:v ratio. The ${}_{w}^{s}\mathrm{pH}$ of the resulting mixture containing 10 mM ammonium acetate and 70% ACN was measured using the pH meter to be 6.7.

### 2.4 Method details

All experiments were carried out at a temperature of 30°C. Mobile phase A consisted of Milli‐Q water, mobile phase B was MS‐grade ACN, and mobile phase D contained aqueous 200 mM ammonium acetate ${}_{w}^{w}\mathrm{pH}$ 5.0. For each experiment, the column was subjected to the following steps. First, the column was equilibrated to the starting conditions (95:5 B:D) for 20.6 min at a flow rate of 0.5 mL/min, equivalent to 30.0 column volumes. Next, a 0.0 μL injection was performed using the gradient outlined below to condition the column and ensure proper post‐gradient re‐equilibration between injections. A gradient of 5–50% aqueous mobile phase was achieved using a constant 5% of mobile phase D to maintain 10 mM buffer concentration throughout the gradient and column equilibration. The composition was held at 95:5 B:D for 2 min, then transitioned linearly to 45:50:5 A:B:D over 4 min. This composition was held for 2 min before returning to 95:5 B:D for re‐equilibration (10 min), representing 14.6 column volumes, a sufficient equilibration according to a recent study [40]. The total run time was 18 min. Next, the 10 samples were injected in sequence and the MS data was used to determine the retention time for each test analyte. The retention times were then entered into a Microsoft Excel Spreadsheet and plotted for all column pairs (plots are provided in Figure S1, retention times in Table S2). We chose gradient experimental conditions to ensure elution of all hydrophobic/hydrophilic analytes in a single experiment. Gradient conditions require column equilibration prior to every experiment. In a recent study, we investigated BEH Amide column equilibration and concluded that equilibration is achieved more rapidly for mobile phases with greater buffer concentration and aqueous content [40]. A higher concentration of water and buffer enables faster buildup of the aqueous layer and equilibration of sorbent ionic sites. This is the case even for zwitterionic HILIC stationary phases that form a thicker aqueous layer than other types of HILIC columns [19, 20, 24]. We choose 14.6 column volumes equilibration conditions for all column types used in this study even though this may result in only partial equilibration of certain HILIC columns. Partial equilibration is sufficient for repeatable gradient experiments [41, 42] and affects the observed retention to a lesser degree than in isocratic elution.

## 3 RESULTS AND DISCUSSION

### 3.1 Comparison of HILIC column selectivity

We investigated the separation selectivity of 19 columns (for description see Table 1) operated in the HILIC mode using 77 polar analytes (see Table S1). The analytes were separated using the gradient described in section 2.4 and the retention times were recorded. The similarity or dissimilarity of any two columns may be estimated by various methods [27, 37, 38, 43–45]. We utilized the selectivity factor s, a modification of the s^2^ parameter proposed by Neue et al. [35]. Selectivity factor s was calculated according to Equation (2), where *r^2^* represents the correlation coefficient for the retention times of a set of analytes on two different columns.

(2) $$s=100\times \sqrt{1-r^{2}}$$

Low s values indicate similar separation selectivity, while the s values approaching 100 signify that the columns have highly different selectivity.

Shown in Figure 1 is a heat map of the s factors for all combinations of the 19 columns. The columns were sorted according to their secondary retention mode (see Table 1). The cyano bonded, bare silica, or hybrid and mixed‐mode HILIC‐1 columns are listed first. These stationary phases exhibit cation‐exchange activity in addition to HILIC retention [24, 37, 38, 46, 47]. This is due to acidic silanols present on the surface of these materials. Next, we list the zwitterionic columns. The stationary phases in these columns are bonded with positively and negatively charged functional groups in a 1:1 molar ratio. While zwitterionic materials should be net neutral, they have been reported to exhibit weak ion‐exchange retention behavior [48, 49]. Next, we list HILIC columns containing stationary phases bonded with neutral groups such as amide, diol, pentahydroxy, or urea. These materials exhibit low ionic retention [46]. Finally, the stationary phases with amines are listed. These columns exhibit anion‐exchange activity in addition to HILIC retention [50].

**FIGURE 2:**
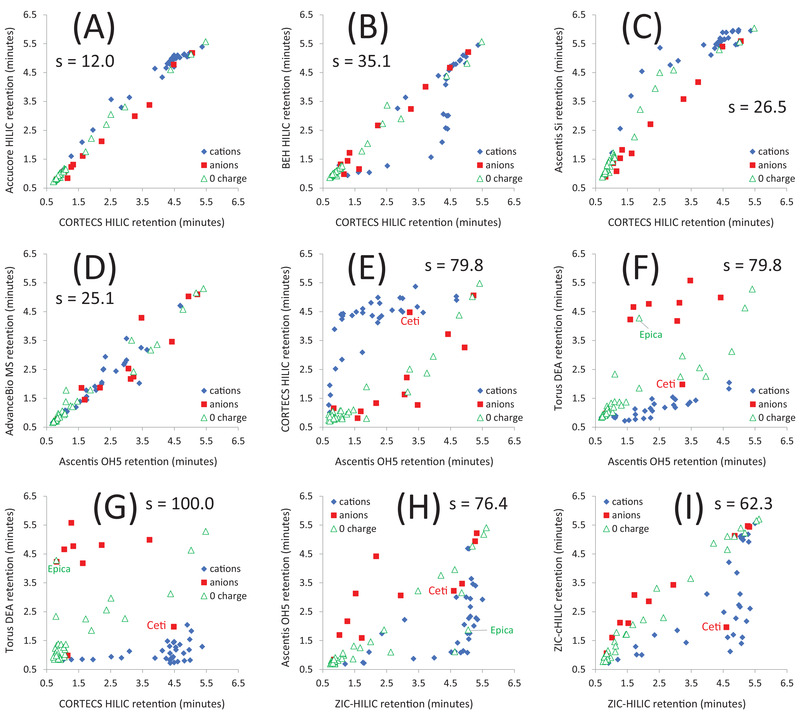
Retention time correlations for selected HILIC columns

The heat map presented in Figure 1 shows that columns packed with similar sorbent chemistries give similar separation selectivities. The s values are low (shaded in red) within the group of silica columns: Ascentis Si, Accucore HILIC, and CORTECS HILIC columns. Similar selectivity of these columns is apparent in Figure 2A,C where the retention data show strong correlations. High column similarity can also be seen in Figure 1 for Kromasil Cyano versus HSS Cyano or Torus Diol versus Ascentis OH5 columns. While low s factors are expected for columns with similar chemistry, we also observed a high degree of similarity for the neutral HILIC stationary phases BEH Amide, Ascentis OH5, and Accucore Urea columns, even though they have different chemistries. The retention plots for these columns are provided in Figure S1.

The values highlighted in green in Figure 1 signify the results with high s values, indicating dissimilar separation selectivities. The highest s values were observed for columns with opposite ion‐exchange activities. This is best illustrated by the example of the CORTECS HILIC (cation‐exchange activity) column versus the Torus DEA (anion‐exchange activity) column, where the s value is 100.

### 3.2 Effect of ACN on analyte charge

The charges of the test analytes depend on the pH of the mobile phase, and their dissociation constants. The p*K*_a_ values for the 77 analytes utilized in our study were obtained from the DrugBank online database/ChemAxon software (Table S1). The charges (*z*) were then calculated according to Shimura et al. [51] for chosen mobile phase pH by summing the charges of the individual ionizable groups of each analyte. The charge calculator is provided as a Supplemental MS Excel spreadsheet “z_calculator.xlsx”.

The limitation of the above‐described approach is that mobile phase pH and p*K*_a_ values of analytes shift in presence of organic solvents; this can alter the apparent analytes’ charge. This phenomenon has been studied by Wiczling et al. [52, 53], Bosch et al. [54, 55], Espinoza et al. [56, 57], and others [58, 59, 60, 61]. Since the HILIC mobile phases include high concentrations of ACN, the calculated analyte charges may be significantly different than those in an aqueous environment. Unfortunately, the p*K*_a_ values of our 77 test analytes in aqueous/ACN mixtures are not known. Therefore, we approximated them based on data for representative small molecules reported in the literature [56, 60]. We estimate that the p*K*_a_ values of acidic functional groups increase on average by 2 units in 70% ACN relative to aqueous conditions and the p*K*_a_ values of basic moieties decrease on average by 1.5 units. We selected an ACN concentration of 70% because it represents approximately the mid‐point of the gradient composition used in our study. We measured a ${}_{w}^{s}\mathrm{pH}$ value of 6.7 for a 70/30 v/v ACN/10 mM ammonium acetate (aq) mixture. We used this value along with the estimated ${}_{w}^{s}p_{\mathrm{Ka}}$ values to calculate the charges for the test compounds in our HILIC experiment (see Table S1).

The proposed correction method is approximate (it neglects the changing ACN concentration in the gradient, and the fact that the p*K*_a_ shifts are not constant for all analytes), but adequate for our purpose. Because the shift in the mobile phase pH (+1.7) was similar to the correction applied for the acid p*K*_a_ values (+2), there was little change in the charge contributed by the acidic functional groups. However, for the basic functional groups, the p*K*_a_ correction (−1.5) is in the opposite direction from the mobile phase pH shift. Consequently, in some cases (when the aqueous p*K*_a_ was between 4.4 – 7.6) there were significant changes in the charge contributed by the basic groups. We classified the analytes in the retention plots as cations for *z* > 0.2, anions for *z* < −0.2 and neutral, or zero charge compounds for *z* between −0.2 to 0.2. The corrections for ACN content resulted in a shift in the categorization from the cation to neutral for eight analytes (adenine, cytosine, 2,6‐dimethylpyridine, ketamine, nalidixic acid, papaverine, pyridine, and pyridoxine). The resulting retention plots presented in Figure 2 and Figure S1 show only a few outliers from the expected trends. This gives us confidence that the retention plots correctly capture the analyte charge classes under our separation conditions. At ${}_{w}^{s}\mathrm{pH}$ = 6.7, 11 analytes were negatively charged, 32 were neutral compounds (near zero charges), and 34 were positively charged analytes.

### 3.3 Impact of electrostatic retention on HILIC separation selectivity

The data summarized in Figure 1 suggest that ionic interactions have a significant impact on HILIC column separation selectivity, in agreement with previous studies [24, 36–38]. This hypothesis is supported by the retention plots presented in Figure 2. We differentiated the cationic (blue diamonds), zero charge (green triangles), and anionic (red squares) analytes to highlight the contribution of electrostatic interactions to analyte retention. Distinct patterns in the behaviors of these three groups of analytes may be observed in many of the plots. For example, it can be seen in Figure 2G that the anions are strongly retained, and the cations are weakly retained on the Torus DEA column. The opposite trends are observed for the CORTECS HILIC column.

In Figure 2A–C we compare the retention behavior of CORTECS HILIC, Accucore HILIC, Ascentis Si, and BEH HILIC columns. All four columns are packed with unmodified silica or hybrid particles with known cation‐exchange retention character (only BEH HILIC is a hybrid silica column, the other three are packed with conventional silica). We expected all plots comparing columns from this group to show high correlations, as is the case in Figure 2A for CORTECS HILIC and Accucore HILIC columns (s = 12.0). However, Figure 2B reveals that the CORTECS HILIC column has stronger retention for several of the cations than the BEH HILIC column, and Figure 2C indicates that the CORTECS HILIC column has weaker retention for several other cations than the Ascentis Si column. Different cation‐exchange activities of silica HILIC columns may be explained by different concentrations, accessibility, and/or acidity of the surface silanols. BEH particles are known to have lower cation exchange activity than conventional silica particles [7, 46, 62].

Figure 2D compares the retention times for the neutral Ascentis OH5 column and the AdvanceBio MS zwitterionic column. No clear differences in the retention of the cations and anions were observed. The different selectivity is attributed not to electrostatic effects, but to other types of interactions such as hydrogen bonding [24, 36–38]. In Figure 2E,F, we compare the retention times for the Ascentis OH5 column to those for the CORTECS HILIC (cation‐exchange activity) and Torus DEA (anion‐exchange activity) columns. Opposite electrostatic retention contributions are observed: the CORTECS HILIC column strongly retains the cations, while the Torus DEA column preferentially retains the anions. The plots in Figure 2E,F show comparably high selectivity differences for the Ascentis OH5 column versus the CORTECS HILIC and Torus DEA columns in terms of the s factors (s = 79.8 for both). However, this does not mean that CORTECS HILIC and Torus DEA columns have similar selectivities. On the contrary, the retention plot for those two columns has the highest dissimilarity of all the column combinations (s = 100.0, Figure 2G).

In Figure 2H we show the retention comparison for the neutral Ascentis OH5 column versus the presumably net neutral zwitterionic ZIC‐HILIC column, while in Figure 2I we compare two zwitterionic columns, ZIC‐HILIC, and ZIC‐cHILIC columns. In both cases, we detected significant differences in the ion‐exchange activity of the columns. The ZIC‐HILIC column showed stronger retention for cations than either the Ascentis OH5 or ZIC‐cHILIC columns. This is in agreement with results reported by Ikegami et al [33].

**FIGURE 3:**
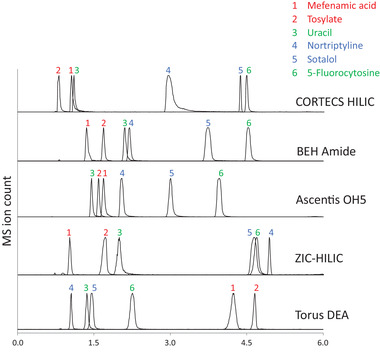
Chromatogram examples for selectedHILIC columns using cationic, neutral, and anionic analytes. The anionic compounds were mefenamic acid (peak 1) and tosylate (peak 2), the neutral compounds were uracil (peak 3) and 5‐fluorocytosine (peak 6) and the cationic compounds were nortriptyline (peak 4) and sotalol (peak 5)

Additional plots provided in Figure S1 reveal that columns with similar chemistry (e.g., Torus Diol vs. Ascentis OH5 or sulfobetaine bonded columns such as ZIC‐HILIC versus BEH Z‐HILIC columns) also appear to have different ion‐exchange activities. This is either due to differences in silanol activity or other factors contributing to the electrostatic activity of the stationary phases. Interestingly, Z‐HILIC and ZIC‐HILIC columns (sulfobetaine columns) are less similar than Z‐HILIC and ZIC‐cHILIC (the latter column has phosphorylcholine chemistry). Figure S1 suggests that electrostatic interactions in HILIC have a stronger impact on the selectivity than the nature of the neutral ligand chemistry (e.g., urea vs. diol or amide). In some cases, the electrostatic retention contribution is so prominent that the separation may be described as mixed‐mode chromatography [46, 50] rather than retention relying only on analyte partitioning between the mobile phase and the aqueous layer on the surface of the stationary phase. This conclusion is illustrated by the chromatograms presented in Figure 3, showing the elution patterns for representative anionic, cationic and neutral analytes on five different columns. A CORTECS HILIC column (cation‐exchange activity) is compared to neutral BEH Amide and Ascentis 5OH columns, a zwitterionic ZIC‐HILIC column, and a Torus DEA column with anion‐exchange activity. Only the neutral BEH Amide and Ascentis OH5 columns show retention selectivity that is not impacted by electrostatic interactions.

It is worth mentioning that when used for HILIC separations, cyano and pentafluorophenyl columns have been reported to retain analytes primarily due to ionic interactions. Both types of the column have negligible HILIC retention for neutral compounds [24, 46]. Supplementary Fig. S1 plots confirm this observation for the cyano columns used in this study.

### 3.4 Analysis of outliers

We argued that ion‐exchange interactions are the strongest factor contributing to the observed column selectivity differences. This finding corroborates previously reported results published by several groups [24, 36–38]. However, close inspection of the plots in Figure 2 and Figure S1 reveals several outliers from the electrostatic retention trends. Two examples are labeled in Figure 2: cetirizine (Ceti) was assigned as an anion but behaves as a cation while the zero charge analyte epicatechin (Epica) behaves like an anion rather than a neutral compound. There are several possible reasons for the observed outliers: (i) experimental or data processing errors leading to analyte misidentification, (ii) a flaw in the estimated ${}_{w}^{s}p$ *K*_a_ values, or (iii) incorrect p*K*_a_ literature data. We acquired the retention data using MS detection, reducing the possibility of data acquisition errors. For this reason, we believe that the p*K*_a_ values calculated by the ChemAxon software and the simplified p*K*_a_ correction method are responsible for the observed outliers.

**FIGURE 4:**
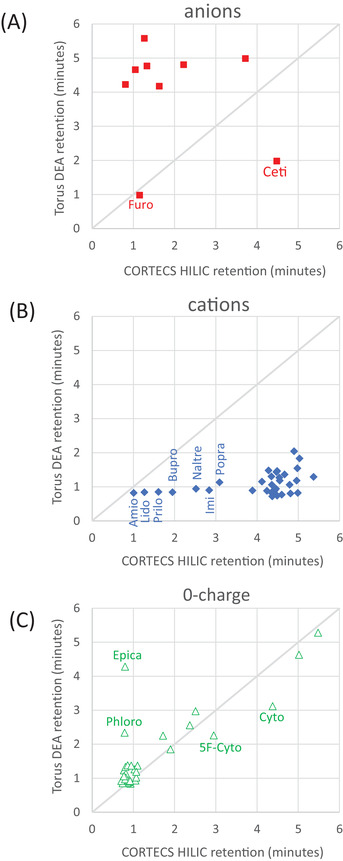
Investigation of outliers in the retention time plots between CORTECS HILIC and Torus DEA columns. (A) anions, (B) cations, and (C) 0 charge analytes are presented in separate plots

In Figure 4 we present a detailed analysis of the retention comparison for the CORTECS HILIC and Torus DEA columns. The anions (Figure 4A), cations (Figure 4B), and zero charge analytes (Figure 4C) are plotted individually. If the columns retain the analytes similarly, the retention data should group along the diagonal. This is indeed the case for the majority of the neutral analytes, which we believe to be retained predominantly by partitioning. However, anions are retained more strongly on the Torus DEA column due to its anion‐exchange activity, resulting in data points located above the diagonal line (Figure 4A). Conversely, the cations, more strongly retained on the CORTECS HILIC column due to its cation‐exchange activity, are located below the diagonal line (Figure 4B).

Several outliers from the expected retention trends are apparent in Figure 4. Ceti, with a calculated charge of −0.65 at the experimental ${}_{w}^{s}\mathrm{pH}$ of 6.7, behaves as a cation. Similar retention trends are seen in Figure 2 for this analyte in other column comparisons. The calculated charge for Furosemide (labeled Furo) at ${}_{w}^{s}\mathrm{pH}$ 6.7 is −0.89, but it can be seen in Figure 4A that its retention follows the trend for the neutral analytes. These outliers may be explained by discrepancies in the estimated ${}_{w}^{s}p$ *K*_a_ values. This is especially likely for Ceti, where the basic p*K*_a_ (6.29) and the acidic p*K*_a_ (5.6) are close to the experimental ${}_{w}^{s}\mathrm{pH}$ of 6.7. Relatively small errors in these p*K*_a_ values or in the estimated ${}_{w}^{s}p$ *K*_a_ values can alter the charge. The same is true for furosemide with an acidic p*K*_a_ of 5.8. We are aware that ChemAxon p*K*_a_ values obtained from the DrugBank database differ in some cases from the most recent version of ChemAxon software p*K*_a_ predictions and values published in the literature. We also know that the uniform p*K*_a_ corrections applied for all acidic and basic groups is an oversimplification, and could well be the cause of the discrepancies for these analytes.

Figure 4B shows that all data points for cations are below the diagonal line. The compounds drifting towards the diagonal line are incompletely charged bases; amiodarone (Amio) with z = 0.65, lidocaine (Lido) with z = 0.26, prilocaine (Prilo) with z = 0.81, and bupropion with z = 0.51. The calculated charges of the remaining cations were close to one except for morphine with z = 1.88.

The analysis of Figure 4C reveals that the data points for cytosine (Cyto) and 5‐fluorocytosine (5F‐Cyto) are situated below the diagonal. On the other hand, phloroglucinol (Phloro) and epicatechin (Epica) are located above the diagonal. The p*K*_a_ values of all these compounds are relatively far from the mobile phase ${}_{w}^{s}\mathrm{pH}$ and all have a charge close to zero. The observed differences in selectivity may be due to hydrogen bond donor/acceptor interactions of the multiple OH groups of phloroglucinol and epicatechin with the Torus DEA Stationary Phase, although other interactions may also contribute to the observed behavior [24, 37, 38].

## 4 CONCLUDING REMARKS

These results show that for samples containing charged analytes, electrostatic interactions are a primary cause of stationary phase selectivity differences in HILIC. These interactions were most prominent for unbonded silica and hybrid materials as well as cyano bonded silicas, which strongly retain cations, and amine‐containing stationary phases, which preferentially retain anions. The greatest selectivity differences (s ranging from 93 to 100) were observed when comparing columns with cation exchange activity to those with anion exchange activity. Comparisons of columns with neutral chemistries (amide, diol, pentahydroxy, and urea) to those with ion‐exchange activity gave somewhat reduced selectivity differences (s between 56 and 99). Smaller differences were observed when comparing only columns with neutral chemistries (s ranging from 28 to 42), while comparisons among zwitterionic columns showed moderate selectivity differences, with s ranging from 35 to 79. These results should be helpful when selecting columns with different selectivity for method development.

Calculating the charges of ionizable analytes under HILIC separation conditions required consideration of the effects of the high mobile phase ACN concentration on the dissociation constants of the buffer and the analytes. We measured the shift in the pH of the ${}_{w}^{w}\mathrm{pH}\;5.0\;$ammonium acetate buffer was used in the mobile phase for the approximate midpoint composition of the gradient (70% ACN), yielding a ${}_{w}^{s}\mathrm{pH}$ value of 6.7. For the analyte p*K*_a_ values, we applied approximate corrections based on previously reported data for representative compounds. Analyte charges based on these corrected values were in better agreement with the retention data than were the charges calculated from the purely aqueous values. Work is in progress to expand this study to mobile phases containing lower and higher pH values; the resulting retention changes of analytes may further support the hypothesis about electrostatic retention participation in HILIC.

## CONFLICT OF INTEREST

The authors are currently employed by Waters Corporation, the manufacturer of several of the columns that were evaluated.

ACQUITY, Atlantis, BEH, CORTECS, QDa, Torus, UPLC, XBridge, Xevo, and XSelect are trademarks of Waters Technologies Corporation. Acclaim and Accucore are trademarks of Thermo Fisher Scientific Corporation. Ascentis, Milli‐Q, SeQuant, and ZIC are trademarks of Merck KGAA. Kromasil is a trademark of Nouryon Pulp and Performance Chemicals. AdvanceBio is a trademark of Agilent Technologies Inc. Microsoft and Excel are trademarks of Microsoft Corporation. All other trademarks are the property of their respective owners.

## Supporting information



Supporting Information

Supporting Information

Supporting Information

Supporting Information

## Data Availability

The data that support the findings of this study are available from the corresponding author upon reasonable request.
