# Supplementary material for: Contribution of ionic interactions to stationary phase selectivity in hydrophilic interaction chromatography
Source: J Sep Sci. 2022 Apr 7;45(17):3264–75. doi: 10.1002/jssc.202200165 (PMC9545918; doi:10.1002/jssc.202200165)

Supplemental Fig. S1: Retention plots correlations for all 19 HILIC columns used in this study. Data are arranged in the same format as in Fig. 1 in the manuscript. For retention times used to construct the plots see Supplemental Table S2.

	S Values	Kromasil Cyano	HSS Cyano	Acclaim MM	Ascentis Si	Accucore HILIC	CORTECS HILIC	BEH HILIC	Obelisc N	ZIC-HILIC	ZIC-cHILIC	BEH Z-HILIC	AdvanceBio MS	Acclaim HILIC	Accucore Urea	BEH Amide	Ascentis OH5	Torus Diol	Torus 2-PIC	Torus DEA
		cation-exchange activity							zwitterionic				neutral						Anion-exchange activity	
cation-exchange activity	Kromasil Cyano																			
	HSS Cyano	16.4																		
	Acclaim MM	41.5	37.5																	
	Ascentis Si	61.6	60.8	55.5																
	Accucore HILIC	58.1	57.1	50.6	17.5															
	CORTECS HILIC	59.7	58.4	48.9	26.5	12.0														
	BEH HILIC	61.0	58.3	38.9	48.0	41.1	35.1													
zwitterionic	Obelisc N	71.8	71.1	62.1	38.6	33.7	31.7	43.9												
	ZIC-HILIC	76.1	75.7	73.0	57.2	52.3	51.8	60.4	44.5											
	ZIC-cHILIC	97.9	97.8	93.7	87.5	85.5	83.6	81.1	71.9	62.3										
	BEH Z-HILIC	99.3	99.1	96.2	92.2	90.6	88.4	84.1	79.0	71.7	35.9									
neutral	AdvanceBio MS	98.0	97.0	92.0	88.5	86.1	82.5	75.5	73.4	74.6	50.8	40.8								
	Acclaim HILIC	87.8	85.2	72.6	71.3	67.7	62.3	47.2	55.3	66.8	62.9	64.3	45.5							
	Accucore Urea	91.8	89.4	79.0	77.1	73.3	67.9	56.6	61.6	71.5	64.3	62.2	39.6	24.4						
	BEH Amide	95.3	94.0	86.5	81.9	79.6	75.5	65.6	67.7	69.6	55.8	48.6	31.3	38.3	37.7					
	Ascentis OH5	97.6	96.3	89.0	86.2	83.9	79.8	71.1	72.2	76.4	58.7	54.0	25.1	36.9	32.9	29.3				
	Torus Diol	98.7	97.3	92.0	89.5	87.7	84.4	77.5	77.9	81.7	67.0	61.3	30.4	48.8	39.1	41.3	27.9			
Anion-exchange activity	Torus 2-PIC	99.5	100.0	98.9	98.4	97.9	96.4	93.2	93.0	92.6	76.3	71.9	53.1	72.7	67.4	67.8	53.9	41.2		
	Torus DEA	96.4	97.3	99.5	99.5	99.7	100.0	99.8	99.8	99.2	89.2	87.2	79.2	91.7	89.3	90.3	79.8	76.5	44.2	

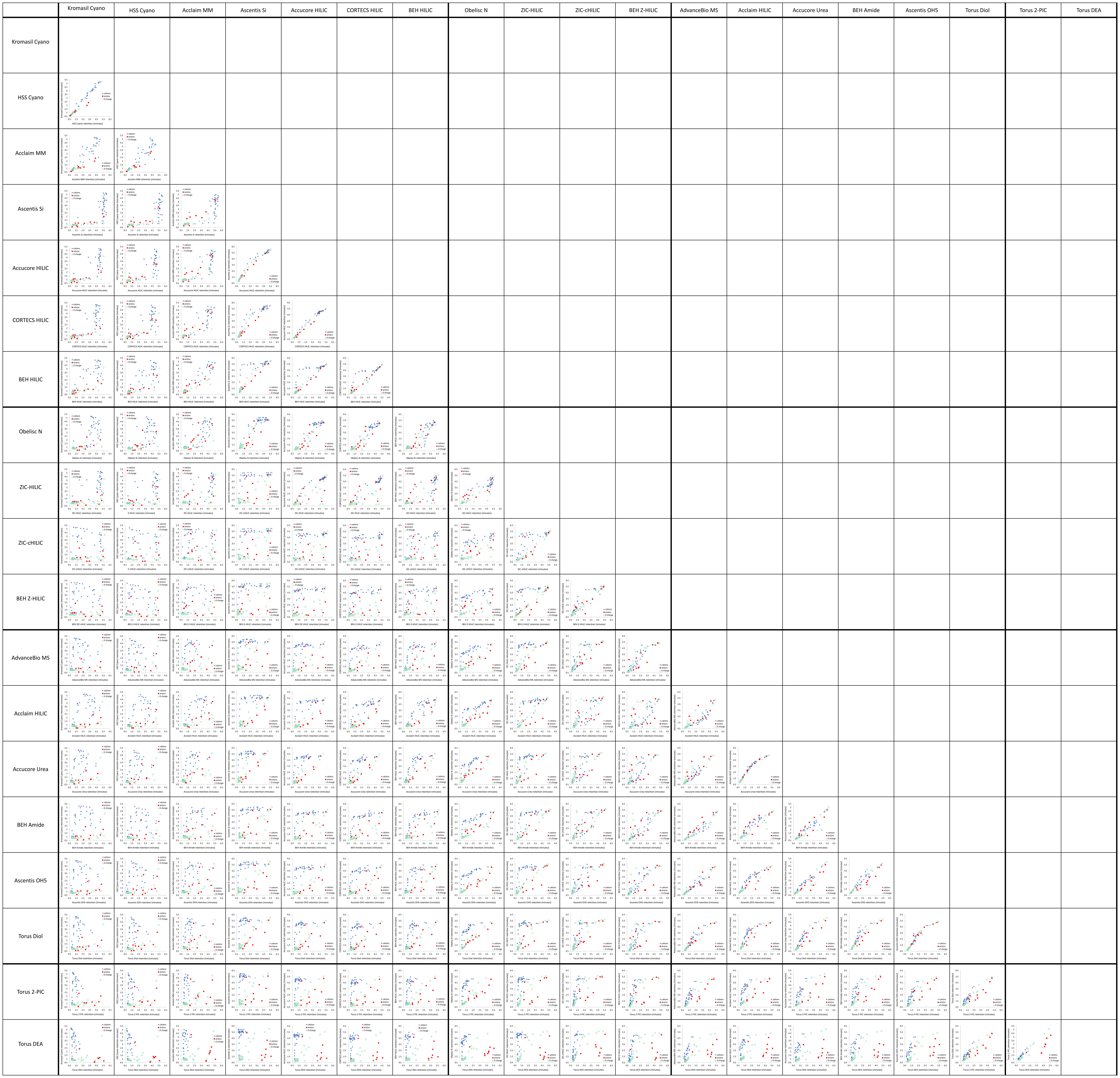

Supplement: Supplementary file 1 — Supporting Information [file JSSC-45-3264-s004.pdf]
